# The Effect of Diluent Mixture with Upper Critical Solution Temperature on Membrane Formation Process, Microstructure, and Performance of PVDF Hollow Fiber Membrane by TIPS Process

**DOI:** 10.3390/polym10070719

**Published:** 2018-06-30

**Authors:** Zhenyu Cui, Shanshan Xu, Jinyue Ding, Jing Zhang, Benqiao He, Hao Wang, Jianxin Li

**Affiliations:** 1 State Key Laboratory of Separation Membranes and Membrane Processes/National Center for International Joint Research on Separation Membranes, School of Material Science and Engineering, Tianjin Polytechnic University, Tianjin 300387, China; xushan3033@gmail.com (S.X.); susanna1ding@163.com (J.D.); hebenqiao@139.com (B.H.); wh576363864@icloud.com (H.W.); 2 School of Computer Science and Software Engineering, Tianjin Polytechnic University, Tianjin 300387, China; jingzhang@tjpu.edu.cn

**Keywords:** thermally induced phase separation, PVDF, microstructure, upper critical solution temperature, interconnection

## Abstract

Thermally induced phase separation (TIPS) is a technique to prepare commercial membrane. However, the quick polymer crystallization during the quenching process will bring about a dense and thick skin layer and thus decrease permeability markedly. In this paper, a diluent mixture with upper critical solution temperature (UCST) was used to prepare polyvinylidene fluoride (PVDF) hollow fiber membrane. That is, the separation between diluent (propylene carbonate (PC)) and non-diluent (dioctyl terephthalate (DOTP)) occurred during the quenching process when the temperature of the dope was lower than 110 °C. The effects of separation between PC and DOTP and the resulting coalescence of DOTP on the PVDF crystallization process, microstructure, and the permeability of the membranes were analyzed. The results showed that the suitable PC/DOTP weight ratio reduced the thickness of the skin layer near the outer surface markedly and resulted in a porous outer surface, and the microstructure evolution process was proposed. The maximum pure water flux for the prepared membrane is up to 128.5 L·m^−2^·h^−1^ even in a dry mode without using a hydrophilizing agent. The rejection rate of the carbonic particle is nearly 100%. This study presents a novel and simple way to fabricate the microporous membrane with the interconnected pore structure.

## 1. Introduction

**Figure 1:**
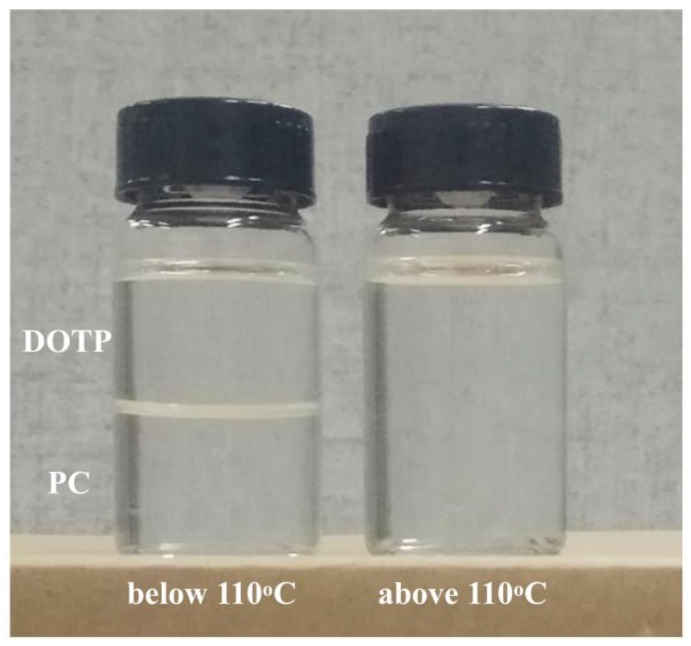
The images of the dioctyl terephthalate (DOTP)/ propylene carbonate (PC) mixture below and above 110 °C.

Thermally induced phase separation (TIPS), based on the heat transfer, is one of the techniques for preparing commercial microporous membrane [1,2,3]. Compared with non-solvent induced phase (NIPS) technique, the membrane via the TIPS had a narrower distribution of pore size, high strength, and ease of regulation of the microstructure [4,5]. Many polymer membranes, such as polyolefin [6,7,8], polysulfone [9], poly(ethylene-*co*-vinylalcohol) (EVOH) [10], polyamide [11], polyphenylene sulfide (PPS) [12], poly(vinyl butyral) (PVB) [13], poly(methy methacrylate) (PMMA) [14], and especially polyvinylidene fluoride (PVDF) [15,16,17] and PVDF-based blends [18,19,20], have been prepared by this technique. Liquid–liquid (L–L) or solid–liquid (S–L) phase separation, relying on the interaction between the polymer and diluent, occurred for the dope during the quenching process [21,22]. In detail, usually a strong interaction between the polymer and diluent brought about the S–L phase separation and formed the spherulites, while a weak interaction led to the L–L phase separation and formed a (closed or somewhat-opened) cellular or network structure [23,24]. During the past few years, some new developments in TIPS technology, such as the combination of TIPS and NIPS [25,26], modified TIPS [27], complex TIPS (c-TIPS) [28], low TIPS (L-TIPS) [29], and reverse TIPS (RTIPS) [30], have been achieved. Even the Janus membrane was developed via the nonsolvent thermally induced phase separation (NTIPS) [31]. The microstructure was regulated by changing the thermodynamics and dynamics of the phase separation for the dope, and the performance of the membrane was improved [24]. The network (formed via spinodal decomposition (SD)), somewhat-opened cellular (formed via nucleation growth (NG) in the metastable region with shorter coarsening time), and spherulitic structures are better than the closed cellular structures (formed via nucleation growth (NG) in the metastable region with longer coarsening time) for the membrane used in the separation field due to the well-interconnected microstructure [24]. Compared with the L–L phase separation, the temperature for the S–L phase separation is much lower, and the membrane formation process is relatively simple. This is because for the L–L phase separation, polymer-lean phase and polymer-rich phase were formed as the dope solution first entered the metastable region, and the coarsening process would go on until the temperature decreased to the crystallization temperature and polymer crystallization started [32]. Conversely, direct polymer crystallization occurred as the temperature of the dope decreased to the crystallization temperature for the S–L phase separation. This suggested that not only could the decomposition of the polymer be avoided, but also the microstructure regulation of the membrane became simple for the S–L phase separation. Up to now, besides a few single diluents, such as diphenyl ketone (DPK, resulting in a somewhat-closed cellular pore in a lower polymer content) and diphenyl carbonate (DPC, resulting in a somewhat-closed cellular pore) [33], most diluents, such as dibutyl phthalate (DBP) [1], glyceryl triacetate (GTA) [21], or propylene carbonate (PC) [27], had a strong interaction with PVDF and thus formed the spherulite. However, the rapid heat transfer between the outer dope and water bath facilitated polymer crystallization and thus resulted in a thick and dense skin layer for the resultant membrane due to the diluent evaporation, which was rejected by the polymer crystallization toward the outer surface of the dope [34]. Slowing down the temperature gradient, for example increasing the temperature of water bath [35] or adding additives, such as solvent (DMAC) into water bath or dope [27,30], or some pore former [36,37], can reduce the thickness of the skin layer and improve the pore interconnection. However, these methods either deteriorated the membrane’s mechanical strength markedly or produced a discarded solution containing multicomponents, which will increase the difficulty and expense of post treatment. The introduction of non-diluent into the polymer/diluent system was another common method to decrease the thickness and increase the interconnection of the skin layer by either weakening the interaction between the diluent and polymer or hindering the PVDF crystallization [34,38,39,40]. However, the thickness of the skin layer can become thinner only with the addition of enough non-diluent leading to the L–L phase separation [41]. As mentioned above, the temperature of the L–L phase separation is usually above the fusion temperature of the polymer [41] and resulted in a somewhat-closed cellular pore structure depending on the coarsening process of the polymer-lean phase [24]. Moreover, the diluent mixture used in the documents was miscible within the experimental temperature range. This suggested that the vacuum distillation had to be employed to separate the higher boiling point mixture of the diluent and non-diluent at a higher temperature after removing the extractant, and this will result in an expensive operating cost. If the diluent mixture with an upper critical solution temperature (UCST) was selected, the separation between the diluent and non-diluent occurred as the temperature decreased to a certain value. It can be conjectured that the separation between them not only omitted the later vacuum distillation operation (the two components of UCST system will automatically divide into two incompatible phases after the extractant was removed and the temperature decreased to the room temperature) and decreased cost, but also restricted the polymer crystallization of the S–L phase separation and more importantly, influenced the coarsening process of the polymer-lean phase and polymer-rich phase for the L–L phase separation by penetrating the boundary between the polymer-lean phase and polymer-rich phase due to the resulting aggregation of each diluent (especially for the less component in the diluent mixture. The less and more component can be regarded as the dispersed phase and continuous phase, respectively. Compared with the continuous phase, the influence of aggregation for the dispersed phase on the coarsening process is more obvious due to the distinct size change of the domain). This suggested that the separation between dioctyl terephthalate (DOTP) and PC and the resulting coalescence of DOTP may be favorable for improving the interconnection of micropore (especially for the thick skin layer) and decreasing filtration resistance. PVDF was insoluble in dioctyl terephthalate at any temperature. This means that PC and DOTP can act as diluent and non-diluent, respectively. Moreover, the authors found that PC and DOTP are only miscible when the temperature is above 110 °C and becomes two phases without emulsification below 110 °C (Figure 1). That is to say, the mixture of PC and DOTP composed the UCST system. However, up to now, no research about the regulation effect of a diluent mixture with UCST on the membrane formation process and membrane microstructure had been reported.

In this paper, the diluent mixture of PC and DOTP with USCT was employed to prepare PVDF hollow fiber membrane via the S–L phase separation of the TIPS process. The aim is to investigate the effects of separation between PC and DOTP and the resulting coalescence of DOTP during the quenching process on the membrane formation process and the microstructure of membrane. In addition, crystallization behavior and the permeability of the resultant membrane were discussed in detail.

## 2. Experimental Section

### 2.1. Materials and Methods

PVDF (Solvay 6020, *M*_w_ = 6.85 × 10^5^) was supplied by Solvay Solexis, Brussels, Belgium. The diluent mixture is composed of PC (analytical reagent) and DOTP (analytical reagent). Bore liquid is DOTP. Anhydrous ethanol was used as extractant to remove DOTP and PC from the nascent membrane. They were all purchased from Tianjin Guangfu Fine Chemical Research Institute (Tianjin, P.R. China). Deionized water was used as the quenching bath. Carbon ink was provided by Ostrich Ink Co., Ltd. (Tianjin, China), and the average diameter size was about 0.16 μm [34].

### 2.2. Phase Diagram of PVDF/PC/DOTP System

Polarizing optical microscopy (BX51C, Olympus, Tokyo, Japan) and differential scanning calorimeter (DSC, 204F1, Selb, Germany) were used to determine the cloud point (*T*_cloud_) and the dynamics crystalline temperature (*T*_c_), respectively. After the dope, composed of the measured PVDF, PC, and DOTP, was quenched in liquid nitrogen, about 5 mg of solidified sample was placed between two cover slips to prevent evaporation, heated to 180 °C on a hot stage (LK-600 THMS, Linkam Scientific Instruments Ltd, Surrey, UK) at the rate of 50 °C·min^−1^, kept at 180 °C for 2 min, and then cooled to 50 °C at the rate of 10 °C·min^−1^. *T*_cloud_ is the temperature corresponding to the appearance of turbidity under the polarizing optical microscopy. Likewise, about 7 mg of solidified sample was sealed in an aluminum pan, heated to 180 °C at the rate of 40 °C·min^−1^, held for 3 min to erase the thermal history, and then cooled to room temperature at the rate of 10 °C·min^−1^. The onset temperature of the exothermic peak was regarded as *T*_c_. Each temperature was tested three times, and the average was calculated.

### 2.3. Preparation of Hollow Fiber Membrane

**Figure 2:**
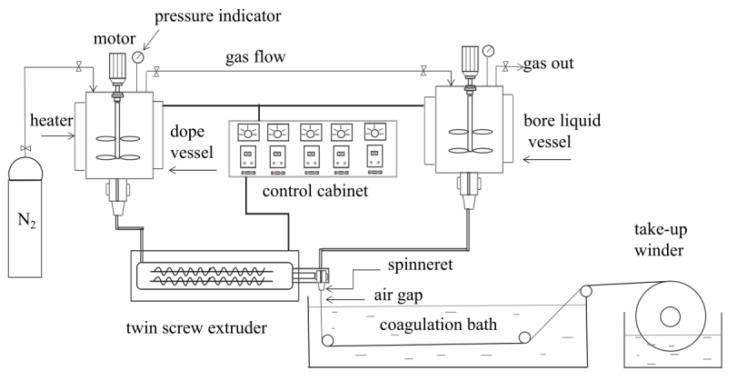
The flow chart for preparing the hollow fiber membrane.

Self-made spinning equipment (Figure 2) was employed to prepare the PVDF hollow fiber membrane. The PVDF content was 25% and the sum of the PC and DOTP was 75%. The weight ratio of PC/DOTP is in the range of 10/0–10/5. The measured PVDF, PC, and DOTP (Table 1) were fed into the vessel and dissolved at 180 °C to form dope. Then, the dope was fed into the twin-screw extruder, extruded from the spinneret at 48 mL·min^−1^ under the metering pump, and wound on a take-up winder at the linear velocity of 20 m·min^−1^ after it was quenched in a water bath (20 °C) to form nascent membrane. The flux of the bore liquid is 24 mL·min^−1^. The diameters of the outer and inner tube for the spinneret were 4 and 2 mm, respectively. The air gap was 0.5 cm. DOTP and the residual PC within the nascent membrane were further extracted by ethanol. The final membrane was dried in air at room temperature and reserved without any post-treatment (for example, being wetted by glycerol aqueous solution).

### 2.4. Characterization of Membrane

The membrane was sputtered with gold after it was freeze-fractured in liquid nitrogen. The surface and cross-sectional images of the membrane were observed by a field-emitting scanning electron microscope (SEM, Sirion-100, FEI Co., Eindhoven, The Netherlands).

DSC was employed to investigate the crystallization behavior of the membrane. Approximately 7 mg of the membrane was weighted and sealed in an aluminum pan. The temperature range was 40–210 °C, and the heating rate was 10 °C min^−1^. The crystallinity (*X*_c_) was calculated using the following Equation (1). Each sample was tested three times, and the average was calculated.

(1) $$X_{c}=\frac{\Delta H_{f}}{\Delta H_{f}^{o}}$$

where Δ*H*_f_^o^, Δ*H_f_* and *X*_c_ represented the PVDF standard fusion enthalpy (100% crystal, 104.7 J·g^−1^ [42]), the fusion enthalpy (J·g^−1^), and the crystallinity (%) of the membrane, respectively.

The crystal structure of the membrane was characterized by X-ray diffraction (XRD D8DIS, COVEX, Karlsruhe, Germany, Cu K_α_ radiation, 40 kV, 30 mA). The scanning speed was 4°·min^−1^. The step size was 0.05°. FTIR (Nicolet, Madison, WI, USA) was used to quantify the content of β-phase. The wave-length range was 700–3500 cm^−1^, and the resolution was 2 cm^−1^. The fraction of β-phase in the membranes was calculated using the following equation [43]:

(2) $$F(\beta )=\frac{A_{\beta }}{1.26A_{\alpha }+A_{\beta }}$$

where *A*_α_ and *A*_β_ are the absorbencies of the α- and β-phase at 764 and 840 cm^−1^, respectively. *F*(β) is the fraction of β-phase.

Porosity was determined by the weighing method and calculated using the following equation [20]:

(3) $$\varepsilon (\%)=\frac{M_{b}/P_{b}}{M_{p}/P_{b}+M_{b}/P_{b}}\times 100$$

where *ε* is the porosity of the membrane. *M*_p_ is the mass of dry membrane. *M*_b_ is the mass of the membrane after absorbing *n*-butanol. *ρ*_p_ and *ρ*_b_ are the density of the membrane and the *n*-butanol, respectively. Each temperature was tested three times, and the average was calculated.

An outside-in operating mode was used to determine the pure water flux (PWF) of the membrane on self-made membrane evaluation equipment. The membrane was pre-pressed at 0.15 MPa for 30 min, and then, the operating pressure decreased to 0.1 MPa, and the permeated water was collected. The PWF was calculated as follows [44]:

(4) $$J_{w}=\frac{V}{A\times t}$$

where *J*_w_ is the PWF (L·m^2^·h^−1^). *A* is the effective outer surface area of the hollow fiber membrane (m^2^). *V* is the volume of permeation (L), and *t* is the running time (h). Each sample was tested three times, and the average was calculated.

The solute rejection was measured at 0.1 MPa in the same apparatus using a suspension system of carbonic ink (0.5 g·L^−1^), and the turbidity of the permeation was measured by turbidimeter (HACH, 2100Q, Loveland, CO, USA). Each temperature was tested three times, and the average was calculated.

## 3. Results and Discussion

### 3.1. The Phase Diagram of the Dope

**Figure 3:**
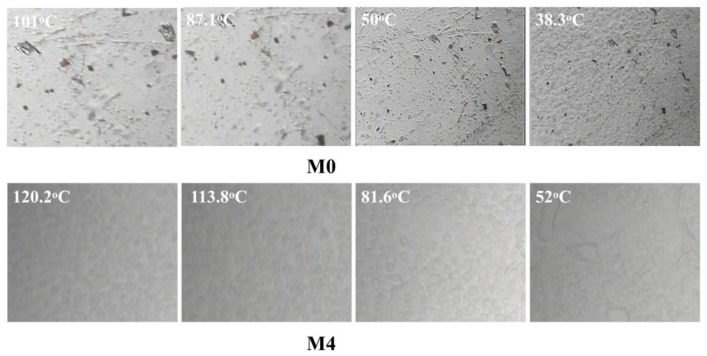
The representative polarizing microscope images of the dope with different the PC/DOTP weight ratios (M0) and (M4).

**Figure 4:**
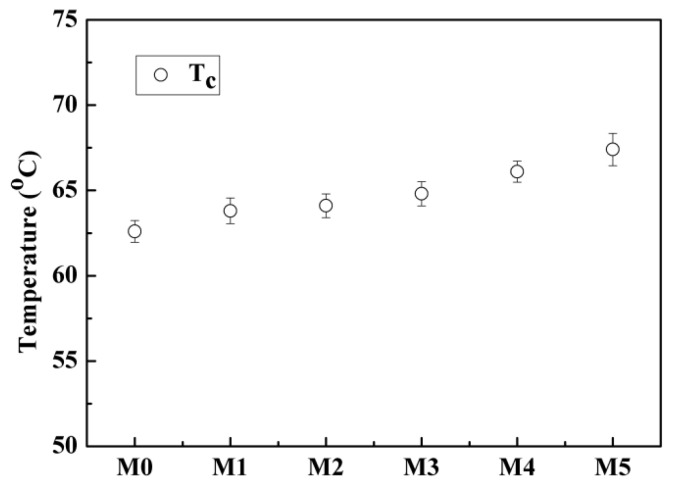
Phase diagram of the PVDF/PC/DOTP system.

For the PVDF/PC system, neither the coarsening process of the polymer-lean phase (the appearance and growth of liquid droplets), nor the nucleation and growth of the PVDF crystallization, can be observed clearly under the polar optical microscope (Figure 3 (M0)) during the cooling process. Only muddy was found when the temperature decreased to about 50 °C, and the muddy became obvious as the temperature further decreased to 38.1 °C. This suggested that the phase separation indeed occurred for the system, and the temperature of solidification and crystallization for PVDF is very low, which can be ascribed to better compatibility between the carbonyl group in PC and fluorine atom in PVDF. Likewise, for the PVDF/PC/DOTP system, neither the coarsening process of polymer-lean phase nor the PVDF crystallization can be observed during the cooling process (Figure 3 (M4)). The liquid appeared when the temperature decreased to about 113.8 °C, and the liquid size became larger. In particular, the temperature decreased to about 52 °C. It should be pointed out that the liquid observed under the polar optical microscope is not the liquid droplets caused by the polymer-lean phase coarsening process of the L–L phase separation. It should be ascribed to the separation between PC and DOTP and the aggregation of DOTP, which covered up the PVDF crystallization and made it invisible under the polar optical microscope. The above phenomena suggested only the S–L phase separation occurred within the whole experimental process and not the L–L phase separation. Although *T*_c_ increased with the increase of the PC/DOTP weight ratio in the phase diagram (Figure 4), the values of *T*_c_ for the dopes are all very low and only a little higher than that of the PVDF/PC system. This meant that the addition of DOTP had less influence on *T*_c_ of the PVDF/PC system. However, the result is reasonable, and the reasons are as follows. No turbidity and crystallization can be observed when the temperature is higher than 110 °C, suggesting that the *T*_c_ for the system is lower than 110 °C. The mass transfer between PC and DOTP should be faster than the PVDF crystallization because of the slower cooling rate (10 °C·min^−1^). This meant that as the temperature decreased to 110 °C and the separation between PVDF and PC occurred, PVDF will dissolve in PC until the temperature further decreased to the crystallization temperature for the PVDF/PC system. This is true of the PVDF crystallization within PC regardless of the addition of DOTP. Finally, the difference of *T*_c_ is not obvious for the different PC/DOTP weight ratios. The slight increase of *T*_c_ with the addition of DOTP can mainly be ascribed to the effect of PVDF content in PC. That is, the higher the PC/DOTP weight ratio, the higher the PVDF content in the PVDF/PC system, which in turn resulted in an easy nucleation of PVDF.

### 3.2. Morphology of the Membrane

**Figure 5:**
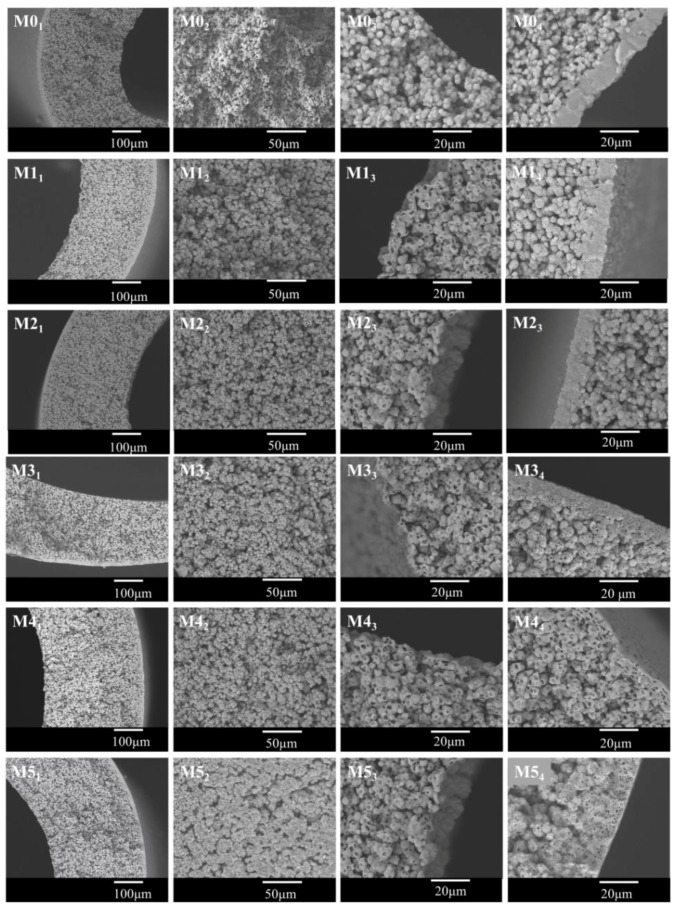
SEM images of the membrane. The whole cross section (MX_1_), magnification of the cross section near the center (MX_2_), cross section near the inner surface (MX_3_), and cross section near the outer surface (MX_4_). X represents the sample code from 0 to 5.

**Figure 6:**
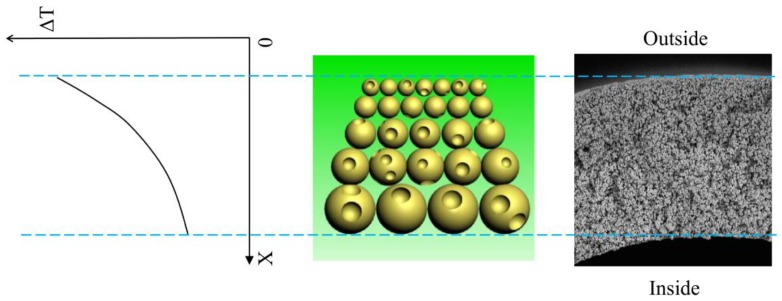
The schematic diagram of the gradient structure formation.

Figure 5 showed the cross-sectional images of the membrane. The spherulites can be observed from all samples, and this further proved that the S–L phase separation occurred for the system during the cooling process. This demonstrated that the quantity of the non-diluent in this paper is not enough to result in a L–L phase separation even if the addition of DOTP will weaken the interaction between PVDF and PC. The asymmetric gradient structure can be found from the whole cross section. That is, there existed a skin layer near the outer surface. The size of the spherulite increased gradually from the outer surface to the inner surface and finally, to no skin layer near the inner surface. This gradient structure could mainly be ascribed to the difference of the temperature gradient, which in turn led to the different crystallization rate for PVDF at different positions of dope. In detail, the difference between the dope and the water bath is much bigger than that between the dope and the bore liquid. Therefore, the heat transfer rate slowed down in sequence from the outer surface to the inner surface, or it corresponded to the decrease of the nucleation rate and facilitated the crystal growth rate from the outer surface to the inner surface. Finally, the faster heat transfer rate led to a dense skin layer or a spherulite of a small size, while the slower heat transfer rate resulted in a spherulite of a big size or a porous skin layer. The schematic diagram of the gradient structure formation is shown in Figure 6. Although there existed the mass transfer between PC and the water bath due to the water solubility of PC, no finger-like pores can be found, and this suggested that compared with TIPS, the influence of NIPS on the bulk membrane structure is negligible, which is different from the reports by Jung [45] and Lee [36]. This may be ascribed to the fact that unlike the PolarClean solvent, PC cannot be soluble with water at any proportion (the solubility is about 240 g·L^−^^1^ at room temperature) and thus cannot bring about an instantaneous demixing of NIPS. Compared with the pristine membrane (M0), the spherulite size became bigger for the membranes using the PC/DOTP mixture. This can mainly be explained from the analysis in Section 3.1, which showed that the PVDF content in PC increased with the higher PC/DOTP weight ratio. The higher polymer content facilitated the growth of crystal and led to a larger spherulite size. Moreover, not only the space between the spherulites increased with the addition of DOTP, but also the spherulites became irregular and some cavities on the surface of the spherulite can be seen (as shown in the higher magnification of the center cross-section in Figure 5). These can mainly be ascribed to the separation between PC and DOTP and the resulting DOTP aggregation. The S–L phase separation process can be depicted as “nucleation-growth” (NG) mode. For the PVDF/PC system of this manuscript, the membrane formation process is the same as that reported in the literature. However, the separation between PC and DOTP occurred as the temperature decreased to 110 °C for the PVDF/PC/DOTP system, and the resulting agglomeration and growth of DOTP are bound to influence the PVDF crystallization process. In detail, the agglomeration and growth of DOTP will occupy the space, which not only enlarged the space between the spherulites, but also hindered the further growth of polymer crystallization and resulted in an irregular spherulite with cavities on the spherulite surface after the aggregated DOTP was extracted. The higher the PC/DOTP weight ratio, the bigger the size of cavity. Finally, the interconnection of the pores was improved.

**Figure 7:**
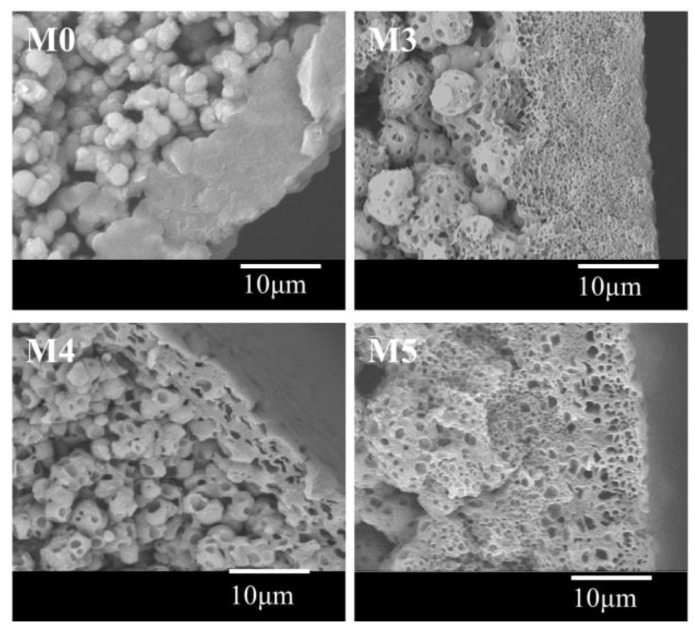
The representative magnification images of the skin layer near the outer surface.

**Figure 8:**
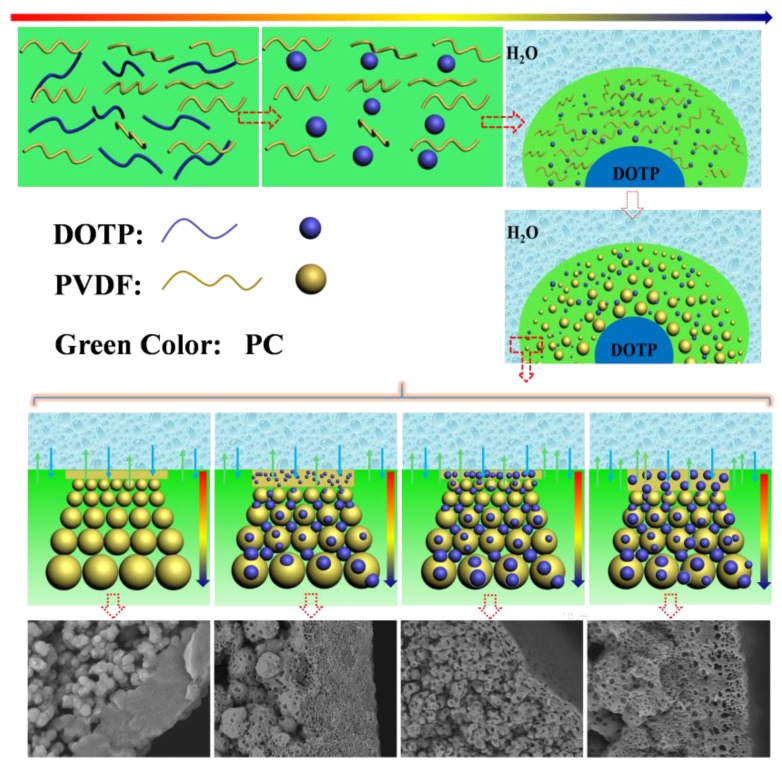
The formation process diagram for the skin layer near the outer surface.

With the addition of DOTP, four different structures for the skin layer near the outer surface (Figure 7) can be seen as follows: (1) the thick and dense skin layer when the PC/DOTP weight ratio is no more than 10/1 (M0 and M1); (2) the loose skin layer with many small size and opened cellular pores when the PC/DOTP weight ratio is in the range of 10/2–10/3 (M2 and M3); (3) the loose cellular pores with thin skin layer for the 10/4 PC/DOTP weight ratio (M4); and (4) the loose skin layer with some big isolated cellular pores as the PC/DOTP weight ratio further reached 10/5 (M5). The change of structure for the skin layer near the outer surface can be ascribed to the comprehensive effects of three factors: the effect of separation between PC and DOTP and the aggregation of DOTP, the diffusion between PC and the water bath, and the evaporation difference of PC and DOTP caused by the elevated spinning temperature. In detail, as reported by Ji et al. [35], the thick and dense skin layer for the pristine PVDF was mainly caused by the evaporation of the diluent near the outer surface at the higher spinning temperature due to the exclusion by the direct polymer crystallization. When the PC/DOTP weight ratio was no more than 10/1 (M1), the influences for both the separation between PC and DOTP and the effect for the resulting penetration of the growth of the PVDF crystallization by the aggregation of DOTP was not obvious because of the lower DOTP content. Therefore, the structure of M1 is similar to that of M0. When the PC/DOTP weight ratio is in the range of 10/2–10/4, for one thing, the elevated spinning temperature not only promoted the mass transfer diffusion between PC and the water bath, but also facilitated the evaporation rate of the diluent. However, the boiling point of DOTP (400 °C) is much higher than that of PC (242 °C), and this suggested that the evaporation rate of PC is much faster than that of DOTP. Therefore, the PC/DOTP weight ratio for the skin layer near the outer surface increased with the increase of DOTP. The higher PC/DOTP weight ratio led to the occurrence of the L–L phase separation and formed the cellular structure for the skin layer near the outer surface. For another, the influence of the separation between PC and DOTP and the resulting aggregation of DOTP became obvious due to the increase of DOTP content, which in turn hindered the coarsening process of the polymer-rich phase and enhanced the penetrating effect to the boundary between the polymer-rich phase and the polymer-lean phase. Finally, the small size and opened cellular pores for the skin layer near the outer surface (M3) and even thin skin layer (M4) was formed. As the PC/DOTP weight ratio further increased to 10/5, the ever higher spinning temperature caused the increased evaporation of PC and much faster diffusion between PC and the water bath, suggesting the further increase of the PC/DOTP weight ratio. Finally, the coarsening process of the polymer-lean phase developed more perfectly and formed a bigger size, creating more somewhat-closed and isolated cellular pores (M5). The formation process diagram for the skin layer near the outer surface is proposed in Figure 8.

**Figure 9:**
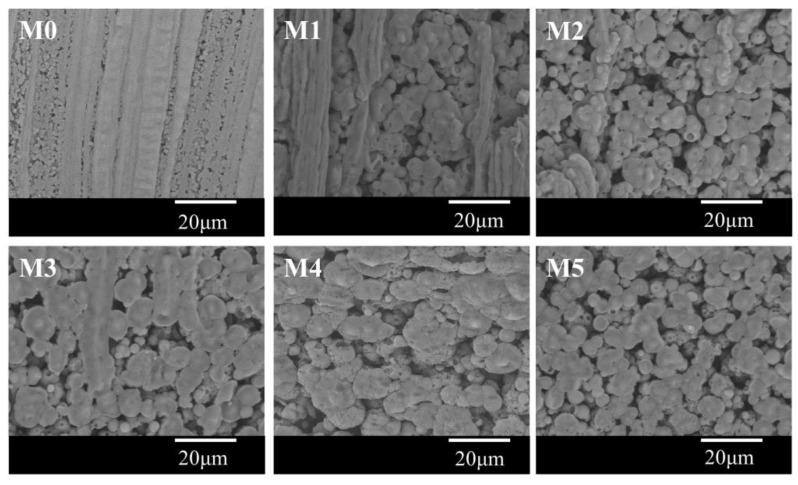
The inner surface image of the membrane.

**Figure 10:**
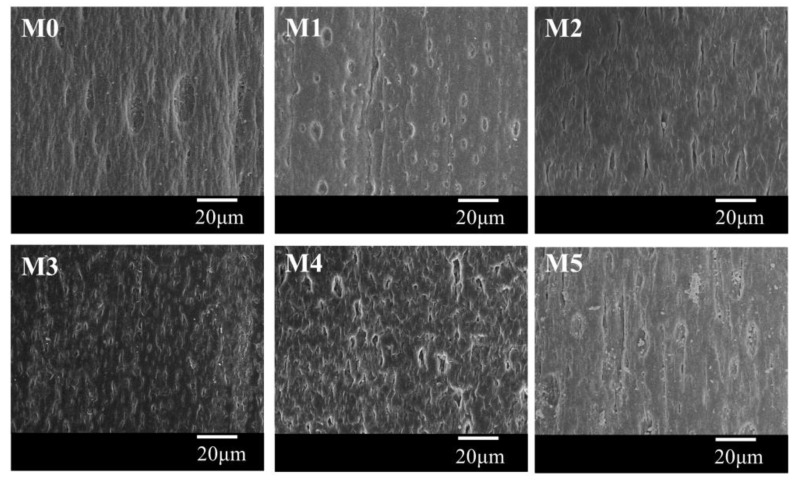
The outer surface image of the membrane.

**Figure 11:**
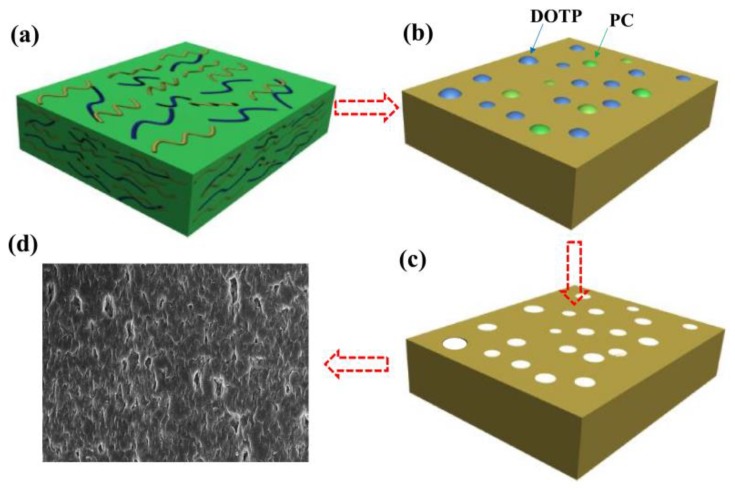
The schematic diagram of the membrane for the porous outer skin layer.

Although the inner surface of all samples is not dense (Figure 9), compared with the pristine membrane, the inner surface for the membranes prepared with the PC/DOTP diluent mixture is more porous. This can mainly be ascribed to the mass transfer between the dope and the bore liquid (the influence of TIPS on the inner surface is the same considering the same temperature difference between the dope and the bore liquid). For the sample of M0, the interfacial temperature between the dope and the bore liquid is less than 110 °C, because the temperature for both of them is less than 110 °C. This meant that no mass transfer occurred between them. Therefore, only the lower temperature gradient resulted in a slower PVDF crystallization and formed a handful of pores within the inner surface. However, for the PC/DOTP diluent mixture, the elevated spinning temperature led to the increase of interfacial temperature between the dope and the bore liquid. The mass transfer between them made the inner surface porous when the interfacial temperature was over 110 °C [35]. Likewise, compared with M0, the outer surface for the membranes prepared by the PC/DOTP diluent mixture were porous, and the pore number increased as the PC/DOTP weight ratio increased from 10/0 to 10/4 (Figure 10). When the PC/DOTP weight ratio further increased to 10/5, the number of pores on the outer surface decreased. The porous outer surface for the membranes prepared by the PC/DOTP diluent mixture was also mainly ascribed to the separation between PC and DOTP and the resulting aggregation of DOTP. This is because according to the mechanism of NIPS and TIPS, the elevated spinning temperature could facilitate double-diffusion between PC and the water bath and form a dense and thick skin layer by the instantaneous phase separation of NIPS. In addition, it can promote the evaporation of the diluent by the increased temperature gradient of TIPS and also form a dense and thick skin layer. The formation process diagram for the outer surface is proposed in Figure 11.

**Figure 12:**
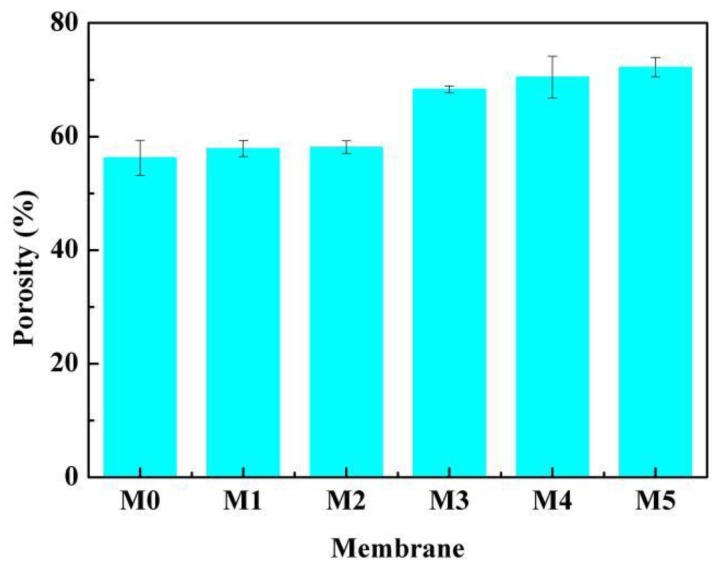
The relationship between porosity and the PC/DOTP weight ratio.

The relationship between the PC/DOTP weight ratio and porosity is shown in Figure 12. It can be seen that porosity increased from 57.9% to 72.2% when the PC/DOTP weight ratio increased from 10/0 to 10/5. This can be ascribed to the fact that the density of PC (1.2 g·L^−^^1^) is larger than that of DOTP (0.984 g·L^−^^1^). That is, the greater the addition of DOTP, the smaller the density or the higher volume of the diluent mixture. Therefore, the higher porosity is achieved after the extraction of the diluent mixture.

### 3.3. Crystallization Behaviors of the Membrane

**Figure 13:**
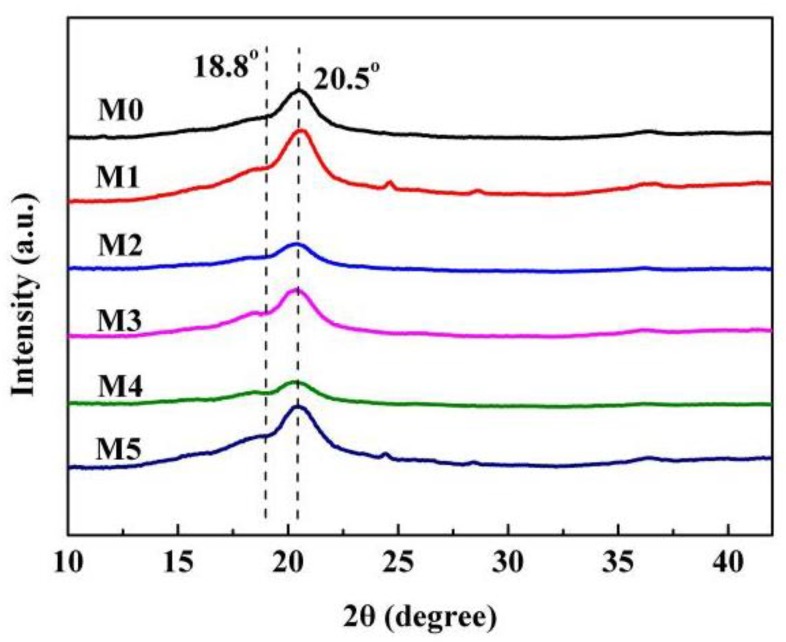
XRD patterns of the membrane.

**Figure 14:**
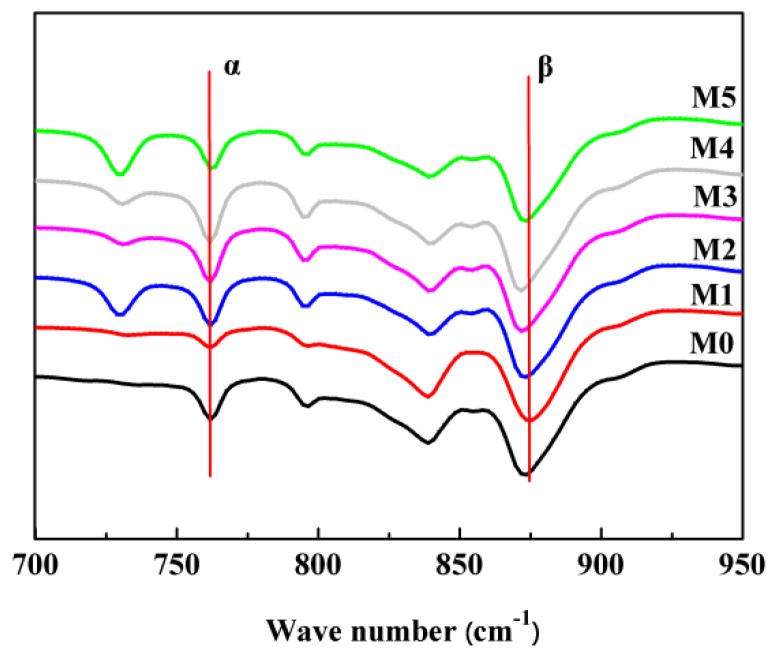
FTIR spectra of the membrane.

**Figure 15:**
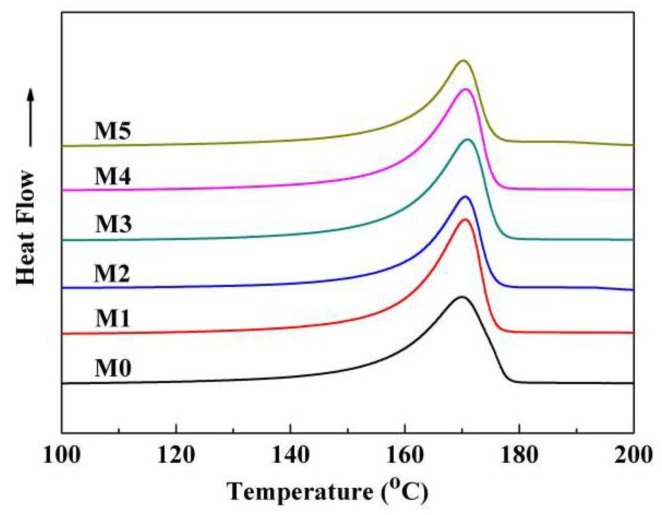
DSC curves of the membrane.

There existed three crystal forms (α, β, and γ) for PVDF, and the α-phase is more common [46]. XRD was used to detect the crystal form of the as-prepared membranes. As shown in Figure 13, the characteristic diffraction peaks at 18.8° and 20.5° corresponded to (020) plate of the α-phase and also corresponded to (110) and (002) plates of the β-phase, respectively [47]. This demonstrated that the as-prepared membranes showed the complex α and β phases. The values of *F*(β), derived from the FTIR measurement (Figure 14) are presented in Table 2. The value of *F*(β) is in the range of 48–57%, and this means that the amounts of α-phase and β-phase are roughly equivalent or that the PC/DOTP weight ratio has less influence on the content of β-phase. This is probably ascribed to the small difference in crystallization temperature (Figure 4), because the content of β-phase was mainly influenced by the crystallization temperature [24]. The fusion enthalpy and the melting peak (*T*_m_^p^, reflecting the thickness of the lamella) obtained from the DSC curves of the membranes (Figure 15) and the calculated crystallinity are listed in Table 3. With the increase of the PC/DOTP weight ratio, the values for both *X*_c_ and *T*_m_^p^ first increased and then decreased, reaching the maximum as the PVDF/DOTP weight ratio became 10/3. This can be ascribed to the comprehensive effects of the following two factors: (1) the PVDF content in PC and (2) the separation between PC and DOTP and resulting aggregation of DOTP. The increased PVDF content helped to increasing *X*_c_, while the separation between PC and DOTP and the resulting aggregation of DOTP hindered the crystallization (reducing *X*_c_) and made the spherulites irregular (reducing the PVDF microcrystal size or the thickness of the lamella). Therefore, the two opposing factors suggested the maximum for both *X*_c_ and *T*_m_^p^ and in a moderate PC/DOTP weight ratio.

### 3.4. Permeability Properties of the Membrane

**Figure 16:**
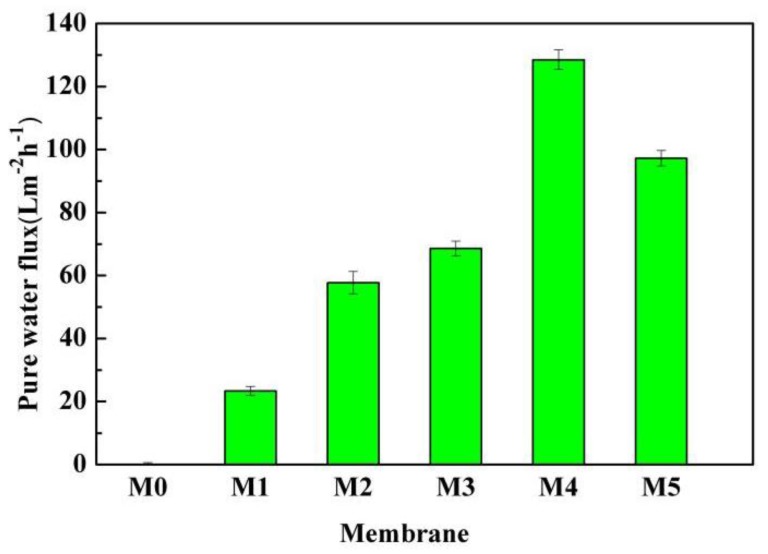
Pure water flux of the membranes (0.1 MPa operating pressure).

Figure 16 indicates the PWF of the membranes. It can be seen that the value of the PWF is near zero for the pristine membrane. However, the PWF increased from 23.2 L·m^−2^·h^−1^ to the maximum of 128.5 L·m^−2^·h^−1^ when the PC/DOTP weight ratio increased from 10/1 (M1) to 10/4 (M4) and then decreased to 97.2 L m^−2^·h^−1^ with the further increase to 10/5 (M5). Two features should be of concern. One is that the as-prepared membranes using the PC/DOTP diluent mixture had a lower filtration resistance, even if the membrane was reserved in a dry mode without any post treatment and the measurement of the PWF was directly conducted without being wetted by a hydrophilic reagent (for example, the PWF is near zero for the dry PVDF membrane without hydrophilization by ethanol [34]). The other is that the PWF is relatively high despite the cellular pores (M3, M4, and M5), and this suggested that the cellular pores in this work are not completely closed. This can mainly be ascribed to the separation between PC and DOTP and the resulting aggregation of DOTP, which in turn hindered the coarsening process of the polymer-lean phase for the skin layer near the outer surface to form a closed cellular pore. The PWF was influenced by the interconnection of pores and porosity. As shown in Figure 5, Figure 6 and Figure 7, for the pristine membrane, the filtration resistance is tremendous because of little pores on the inner and outer surfaces and the thick and dense skin layer formed via the S–L phase separation similar to the reports by Ji et al. [34] and Cha et al. [27]. However, for the membranes prepared by the PC/DOTP diluent mixture, there not only existed some pores on the inner and outer surfaces and the larger space among the spherulites, but also the skin layer became porous and interconnected when the PC/DOTP weight ratio increased from 10/1 to 10/4. That is, the interconnection for the membranes prepared by the diluent mixture with USCT is better. In addition, porosity also increased with the addition of DOTP. These all meant that the filtration resistance decreased and resulted in a higher flux. Compared with the larger, somewhat-closed, and isolated cellular pores of M5, the thin skin layer of M4 is much more interconnected due to lots of smaller, opened cellular pores. Therefore, the PWF is higher.

**Figure 17:**
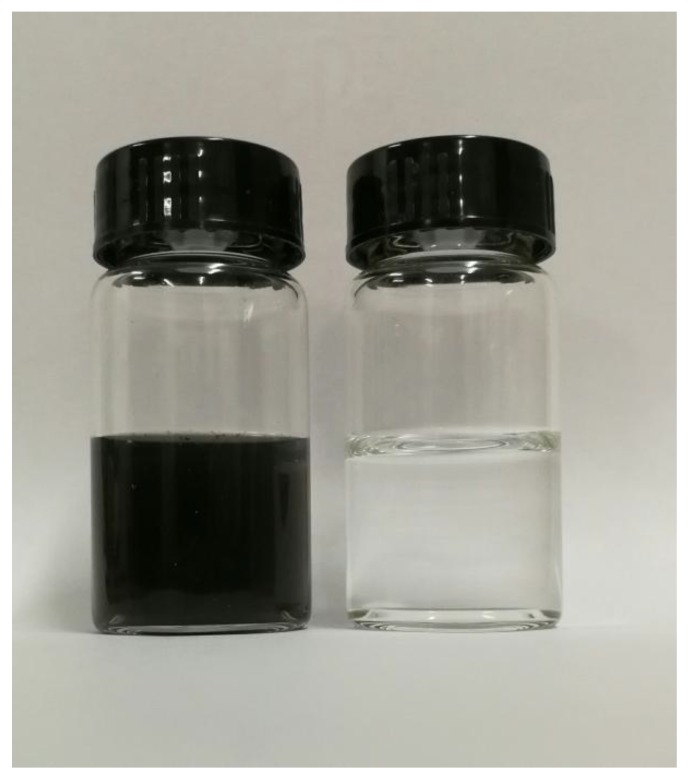
The feed (left) and permeating solution (right) obtained from M4.

The carbon ink suspension was selected to test the particle rejection of the membrane (M4). It can be seen from Figure 17 that the permeating solution is transparent compared with the feed and the turbidity is nearly zero. This indicated that the rejection of the carbon black particles is nearly 100%, which indicated a better rejection capability.

## 4. Conclusions

The diluent mixture system with UCST was firstly introduced to prepare the PVDF hollow fiber membrane via the S–L phase separation of the TIPS process. The separation between PC and DOTP and the resulting aggregation of DOTP not only influenced the polymer crystallization process of the bulk dope, the coarsening process polymer-rich phase, and the polymer-lean phase for the dope near the outer surface, but can also penetrate the boundary between the polymer-rich phase and the polymer-lean phase, which in turn led to a membrane with a porous outer surface and decreased the thickness of skin layer near the outer surface. Finally, a membrane with an interconnected structure was obtained. The maximum PWF of the dry membrane reached 128.5 L m^−2^·h^−1^ without being wetted by a hydrophilizing agent in advance. The rejection rate of the carbonic particle was nearly 100%. In summary, our study provides a new avenue to tailor the micropore structure of a hollow fiber membrane with improved interconnection during the TIPS process.

## Figures and Tables

**Table 1:** Code of membrane, PC/DOTP weight ratio, and spinning and bore liquid temperature.

Code	PC/DOTP	Spinning temperature	Bore liquid temperature
	(*wt*./*wt*.)	(°C)	(°C)
M0	10/0	100	70
M1	10/1	120	90
M2	10/2	130	100
M3	10/3	150	120
M4	10/4	170	130
M5	10/5	180	140

**Table 2:** *A*_α_, *A*_β_, and *F*(β) values of the membranes.

Samples	M0	M1	M2	M3	M4	M5
*A*_α_	1963.2	1398.9	1484.6	1614.0	1753.0	1361.4
*A*_β_	2537.5	2334.2	2138.6	2064.8	2085.2	2064.5
*F*(β) (%)	51.0	57.0	53.4	50.3	48.0	54.6

**Table 3:** The values of melting peak temperature, fusion, and crystallinity.

Samples	M0	M1	M2	M3	M4	M5
*T*_m_^p^ (°C)	170.0	170.5	170.6	170.8	170.6	170.2
Δ*H*_f_ (J g^−1^)	71.45	72.17	72.98	75.52	69.05	63.43
*X*_c_ (%)	68.24	68.93	69.70	72.13	65.95	60.58
